# Antagonist of the neurokinin-1 receptor curbs neuroinflammation in ex vivo and in vitro models of Lyme neuroborreliosis

**DOI:** 10.1186/s12974-015-0453-y

**Published:** 2015-12-30

**Authors:** Alejandra N. Martinez, Geeta Ramesh, Mary B. Jacobs, Mario T. Philipp

**Affiliations:** Division of Bacteriology & Parasitology, Tulane National Primate Research Center, Covington, LA USA; Department of Microbiology and Immunology, Tulane University Medical School, New Orleans, LA USA

**Keywords:** Lyme neuroborreliosis, *Borrelia burgdorferi*, Substance P, NK_1_R antagonist, CCL2, IL-6, CXCL8, Apoptosis

## Abstract

**Background:**

Lyme neuroborreliosis (LNB) can affect both the peripheral (PNS) and the central nervous systems (CNS); it is caused by the spirochete *Borrelia burgdorferi*. The neuropeptide substance P (SP) is an important mediator of both neuroinflammation and blood-brain barrier dysfunction, through its NK_1_ receptor. Increased levels of SP have been shown to correlate with cell death. The present study used both ex vivo and in vitro models of experimentation to determine if the inflammatory mediator production and concomitant cell death caused by exposure of neural tissues and cells to *B. burgdorferi* could be attenuated by treatment with a NK_1_ receptor antagonist.

**Methods:**

We incubated normal rhesus frontal cortex tissue explants (CNS) and primary cultures of rhesus dorsal root ganglia cells (PNS) with live *B. burgdorferi* and tested the effectiveness of the NK_1_ receptor antagonist L703,606 in attenuating inflammatory immune responses and neuronal and glial damage. Culture supernatants and tissue lysates were subjected to multiplex ELISA to quantify immune mediators, while the cells were evaluated for apoptosis by the in situ TUNEL assay. In addition, we identified immune mediators and producer cells in tissue sections by immunofluorescence staining and confocal microscopy.

**Results:**

Co-incubation of both CNS tissues and PNS cells with the NK_1_ receptor antagonist attenuated bacterially induced increases in inflammatory cytokine and chemokine production, particularly, IL-6, CXCL8, and CCL2, and reduced apoptosis levels. Confocal microscopy confirmed that neurons and glial cells are sources of these immune mediators. These results suggest that NK_1_R antagonist treatment is able to reduce downstream pro-inflammatory signaling, thereby indicating that its systemic administration may slow disease progression.

**Conclusions:**

We propose that SP contributes to neurogenic inflammation in LNB, and provide data to suggest that an NK_1_ receptor antagonist may represent a novel neuroprotective therapy.

## Background

Inflammation caused by the spirochete *Borrelia burgdorferi* is an important factor in the pathogenesis of Lyme neuroborreliosis (LNB) [1]. This form of Lyme disease, which can affect both the central (CNS) and peripheral nervous systems (PNS), manifests in 10–15 % of untreated patients [2]. The invasion of the CNS by *B. burgdorferi* can lead to increased levels of pro-inflammatory molecules such as IL-6, IL-12, IL-18, and IFN-γ, and the chemokines CXCL8, CCL2, CXCL11, and CXCL13 [3, 4]. Previously, our laboratory demonstrated that interaction of *B. burgdorferi* with tissue sections isolated from rhesus brain parenchyma and cultured ex vivo induces inflammatory mediators in glial cells, as well as oligodendrocyte and neuronal apoptosis [5]. We also showed that co-culture in vitro of *B. burgdorferi* with cells isolated from rhesus dorsal root ganglia (chiefly neurons) elicited pro-inflammatory mediators from these cells and caused neuronal apoptosis [6]. Moreover, when neurons of a neuronal cell line were incubated with *B. burgdorferi*, the neurons died by apoptosis, but only when purified rhesus microglia were also present [7]. Microglia are potent mediators of CNS inflammation [7] as they act as the primary sensors of danger signals or altered microenvironment.

Substance P (SP) is an 11-amino acid neuropeptide and the most abundant member of the tachykinin family of neuropeptides. SP originates from several cellular sources such as neurons, endothelial cells, and immunocytes, and is released by peripheral nerve endings and central terminals of sensory neurons in the CNS [8]. The biological responses to SP are mediated by the neurokinin-1 receptor (NK_1_R), a G-protein-coupled receptor bearing seven transmembrane domains [9]. Previous studies have shown that SP can synergistically augment *B. burgdorferi*-induced expression of COX-2 in murine microglia [10], and that endogenous SP/NK_1_R interactions are required for maximal inflammatory responses to in vivo challenge with bacteria such as *Neisseria meningitidis* or *B. burgdorferi* [11]. Furthermore, systemic administration of a specific NK_1_R antagonist (L703,606) significantly reduced CNS gliosis, demyelination, and associated inflammatory cytokine elevations in murine models of bacterial meningitis [11].

In view of these results, obtained with murine models, we wished to test the effectiveness of this NK_1_R antagonist (L703,606), in tissues and cells of an animal model that, unlike the mouse, reproduces all of the signs of Lyme disease, including neuroborreliosis [12–14]. We tested if inhibition of SP/NK_1_R interactions was effective in attenuating inflammatory immune responses and neuronal and glial damage in a non-human primate (NHP) cortical brain explant ex vivo culture model of *B. burgdorferi* CNS infection, as well as in primary cultures of dorsal root ganglia (DRG) cells from normal adult rhesus macaques, as an in vitro model of PNS infection. The demonstration that inhibition of SP/NK_1_R interactions ameliorate acute bacterially induced damage in NHP cortical brain tissue and in PNS neurons is a significant step in showing that such an approach could be effective as adjuvant therapy in the context of antibiotic treatment, to limit neuroinflammation and neurologic damage in conditions such as bacterial meningitis.

## Methods

### Brain tissues

Frontal cortex tissues for ex vivo experiments were collected from seven rhesus macaques (*Macaca mulatta)* that were slated for euthanasia because they had chronic idiopathic diarrhea or had undergone trauma. Animals were euthanized by a method consistent with the recommendations of the American Veterinary Medical Association’s Panel on Euthanasia.

### Incubation of brain slices with *B. burgdorferi,* and NK_1_R antagonist treatment

Freshly collected brain tissue was obtained from the frontal cortex immediately after euthanasia. The tissue was sliced into 2-mm sections, and each section was placed in separate wells of 12-well plates. Each well contained 2 mL of RPMI 1640 medium (BioWhittaker, Walkersville, MD) supplemented with 10 % FBS, as previously described [5]. Tissue sections were exposed to medium alone or to medium with added *B. burgdorferi* strain B31 clone 5A19 spirochetes (1 × 10^7^ bacteria/mL) in the presence or absence of 100 μM NK_1_R antagonist (L-703,606 oxalate salt hydrate, Sigma-Aldrich, St. Louis, MO). The wells that received NK_1_R antagonist were pre-treated for 2 h prior to the addition of spirochetes, or medium alone. Incubation at 37 °C for 4 h was allowed to proceed in a humidified 5 % CO_2_ incubator. At the end of the incubation period, half of the total number of tissue slices was fixed in 2 % paraformaldehyde and cryopreserved as described earlier [6]. The other half was processed to obtain protein lysates, and supernatants from whole sections, as described below.

For tissue protein extraction, a ratio of tissue to CelLytic MT reagent of 1:20 (1 g of tissue/20 mL of reagent) containing Protease Inhibitor Cocktail (Sigma) to a final dilution of 1:400 was added to gentleMACS™ M tubes (Miltenyi BioTec, San Diego, CA). Tissues were lysed in a single run using the Protein 1 setting of a gentleMACS™ Dissociator (Miltenyi BioTec) for 53 s and cooled on ice for 2 min. The lysed tissue was centrifuged at 3273 ×*g* for 15 min at 4 °C to pellet the tissue debris. The protein-containing lysate was decanted and stored at −80 °C.

### Immune mediators in ex vivo culture supernatants and lysates

The concentrations of cytokines and chemokines present in the tissue slice culture supernatants and lysates were quantified using the MILLIPLEX MAP Non-Human Primate Cytokine Magnetic Bead Panel - Premixed 23 Plex, PCYTMG-40 K-PX23 Cytokine-Chemokine Array kit (Millipore, Billerica, MA) following the manufacturer’s instructions.

### Immunofluorescence staining for detection of intracytoplasmic immune mediators

For in situ analysis of intracytoplasmic immune mediators, frozen tissue blocks were cryosectioned into 16-μm sections as previously described [6]. Briefly, permeabilization and blocking were performed with 0.1 % Triton X-100-PBS-0.2 % fish-skin gelatin for 30 min, followed by additional blocking incubation with 10 % goat serum-PBS-0.2 % fish-skin gelatin for 1 h. The primary antibodies that were used to label various cell phenotypic markers were anti-human 2′,3′-cyclic nucleotide 3′-phosphodiesterase (CNPase), clone 11-5B mouse IgG1 (Millipore) at 10 μg/mL, anti-human S-100 (Sigma) at 1:500, anti-human neuronal protein NeuN, MAB 377 clone A60, mouse IgG1 (Millipore) at 1:10, anti-human glial fibrillary acidic protein (GFAP), at 1:200, clone G-A-5 purified mouse immunoglobulin conjugated to Cy3 (Sigma), and 2 μg/mL of chicken polyclonal anti-human IBA1 antibody (Aves Labs, Inc., Tigard, OR). Primary antibodies for immune mediators were either anti-human IL-6, mouse IgG2a at 1:1000 (ProSpec, Ness Ziona, Israel), anti-human CCL2 rabbit polyclonal IgG ab7814 at 1:50 (AbCam, Cambridge, MA), or anti-human CXCL8 rabbit polyclonal IgG at 10 μg/mL (RDI, Flanders, NJ). Isotype controls (Sigma) at the concentrations of the respective primary monoclonal antibodies and universal rabbit negative control (Dako Cytomation, Carpinteria, CA) for rabbit polyclonals were also included. Incubation with the primary antibody was followed by secondary antibody staining conjugated to Alexa 488-FITC (green), Alexa 633 (far red), or Alexa 568 (red) (Molecular Probes, Life Technology, Inc. Grand Island, NY). Samples were analyzed on a Leica DMi8 confocal microscope equipped with three lasers (Leica Microsystem, Exton, PA).

### Incubation of DRG cell cultures with *B. burgdorferi* and NK_1_R antagonist treatment

Chamber slides (two wells) with detachable culture slides were first coated with poly-D lysine (BD Biosciences, Franklin Lakes, NJ) and then laminin (Invitrogen) at a final concentration of 10 μg/mL for a minimum of 2 h before seeding the cells. Before plating the DRG cells, the laminin was removed. DRG were obtained from two adult rhesus macaques at necropsy, minced, trypsinized in 5 mL of 0.25 % trypsin-EDTA (Invitrogen) with 1000 units of DNAse (Sigma), pelleted and seeded at 1 × 10^5^ cells per well. DRG cell cultures were maintained for a period of 6 to 7 days in complete DMEM F12 medium containing 10 % FBS and 1X penicillin/streptomycin (P/S) supplemented with fresh L-glutamine (2 mM), and NGF-7S (50 ng/mL, Invitrogen) (complete medium). The DRG cell culture protocol has been thoroughly described previously [6].

DRG cell cultures were pre-incubated in complete medium as above but without P/S, and treated with 10 μM of NK_1_R antagonist (L-703,606 oxalate salt hydrate, Sigma) for 2 h at 37 °C, or left untreated. Cultures were then stimulated with live *B. burgdorferi* at a multiplicity of infection (MOI) of 10:1 at 37 °C for 24 h. After 24 h, culture supernatants were collected and processed for quantification of inflammatory mediators, and cells were fixed and evaluated for apoptosis by the TUNEL assay as described below. Medium controls that were pre-treated and then incubated with the same respective concentrations of NK_1_R antagonist but without the addition of live *B. burgdorferi* were also included.

### Apoptosis by in situ TUNEL assay

Tissue slides as well as DRG cell culture chamber slides were incubated with anti-NeuN or anti-S-100 antibodies prior to performing the TUNEL assay. Slides were then subjected to the TUNEL-ApopTagPlus fluorescein in situ apoptosis assay (Chemicon, Temecula, CA) as per the manufacturer’s instructions. The percentage of apoptotic neurons in brain sections and DRG cell cultures, or the percentage of apoptotic oligodendrocytes in brain sections, was evaluated by counting at least 500 cells in ten microscope fields, followed by the percentage of cells that showed co-localization of both the TUNEL signal and NeuN or S-100 expression. Cells were counted by viewing slides under a fixed magnification of 40x using Nuance Multispectral Imaging System (CRi, PerkinElmer, Waltham, MA). The identity of the oligodendrocytes that stained with the anti-S-100 antibody was confirmed by their morphology.

### Statistical evaluation

The unpaired-two tailed *t* test was used to evaluate the statistical significance between means of data sets, using Graphpad Prizm software (Graph Pad Software Inc., La Jolla, CA) version 5a. A *p* value of 0.05 or lower was considered to be statistically significant.

## Results

### Analysis of SP/NK_1_R interactions in the ex vivo model of CNS infection

#### Quantification of pro-inflammatory mediators

**Fig. 1:**
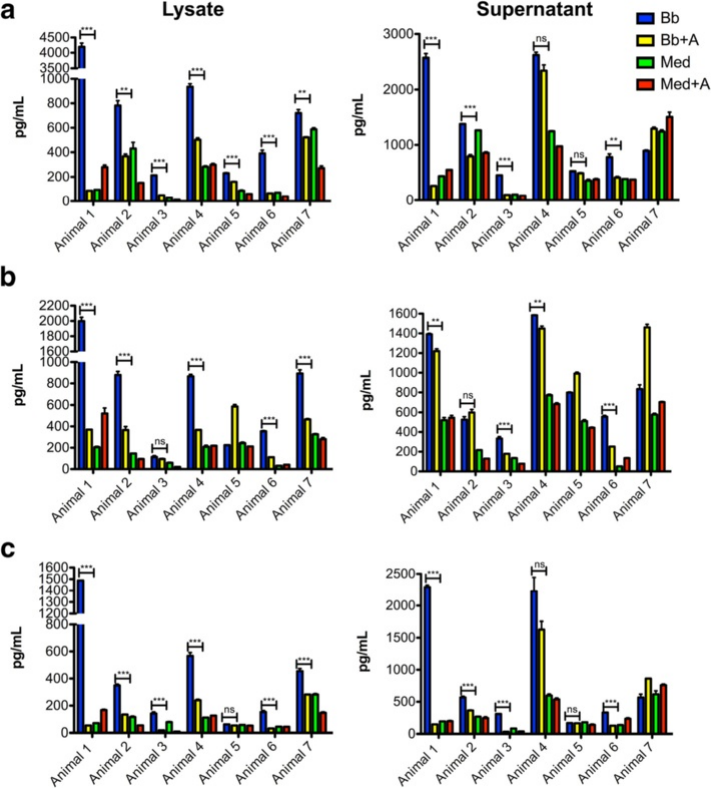
Pro-inflammatory mediator levels in the supernatants and lysates of rhesus macaque frontal cortex sections cultured ex vivo. IL-6 (**a**), CXCL8 (**b**), and CCL2 (**c**) levels were determined by multiplex ELISA in brain tissues that were exposed to *B. burgdorferi* (Bb), medium alone (Med), *B. burgdorferi* plus NK_1_R antagonist (Bb + A), or medium alone plus antagonist (Med + A). The figure shows the arithmetic means ± standard deviations of experimental replicates for each individual animal. **p < 0.001, ***p < 0.0001, *ns* not significant

Interaction of *B. burgdorferi* with rhesus macaque brain parenchyma sections cultured ex vivo elicited IL-6, CXCL8, and CCL2 production that was detectable in both lysate and supernatant, as determined by the NHP Cytokine 23 BioPlex Panel. The addition of NK_1_R antagonist significantly reduced these inflammatory cytokine levels, albeit with animal-to-animal variations (Fig. 1). This pattern of response was seen both in the tissue lysates and tissue section supernatants. It suggests that SP receptor antagonist is able to significantly reduce inflammation derived from the presence of *B. burgdorferi* within the CNS.

#### Quantification of apoptosis

**Fig. 2:**
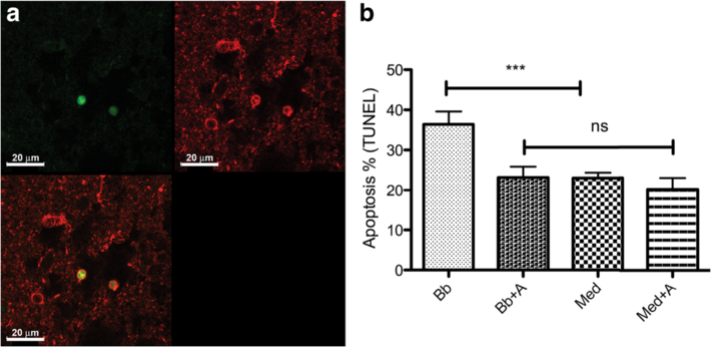
Neural cell apoptosis in rhesus frontal cortex tissue sections cultured ex vivo. **a** S-100-positive cells (oligodendrocytes, as identified by morphology) and DNA fragmentation detected by the TUNEL assay in green, as visualized by fluorescent microscopy. **b** Graphical representation of the percent apoptosis as detected by the TUNEL assay in frontal cortex tissue exposed for 4 h to *B. burgdorferi* (Bb), exposed to *B. burgdorferi* and treated with NK_1_R antagonist (Bb + A), in medium alone (Med), and medium with added NK_1_R antagonist (Med + A) (***p < 0.0001, ns not significant)

We next evaluated the likely impact of *B. burgdorferi-*induced inflammation in the induction of apoptosis. As in previous studies [1], we found oligodendrocytes undergoing apoptosis (Fig. 2). Importantly, there was a significant reduction in the amount of *B. burgdorferi*-induced apoptosis in the presence of NK_1_R antagonist.

Neuronal cell death was also evaluated, and overall, the level of neuronal apoptosis was much lower than that seen in oligodendrocytes (less than 2 % in frontal cortex tissues exposed *to B. burgdorferi* for 4 h). In addition, there were no significant differences in the reduction in the level of *B. burgdorferi*-induced apoptosis in the presence of NK_1_R antagonist (data not shown).

#### Cellular sources of pro-inflammatory mediators

**Fig. 3:**
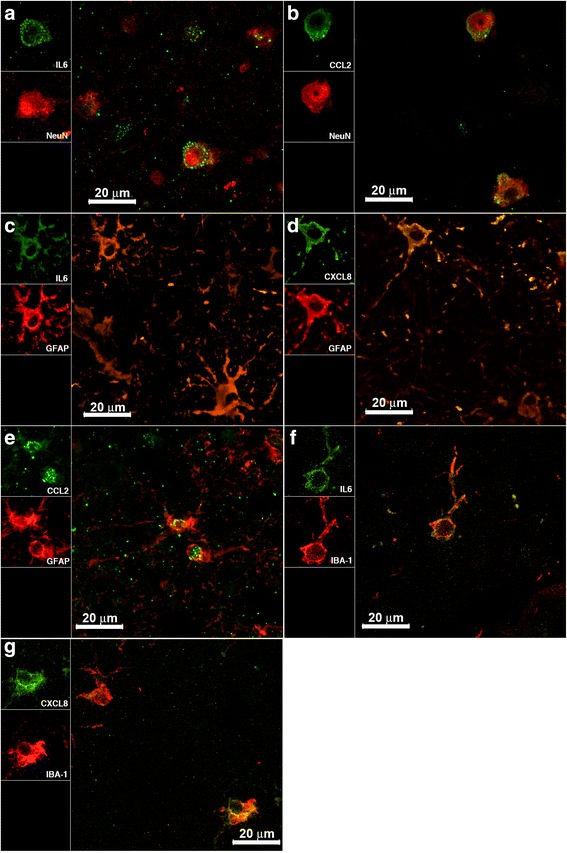
Visualization of IL-6, CXCL8, and CCL2. **a** IL-6 (*green*) inside neurons stained red with anti-NeuN antibody. **b** CCL2 (*green*) inside neurons that were stained red with anti-NeuN antibody. **c** IL-6 (*green*) and astrocytes stained red with anti-GFAP antibody. **d** CXCL8 (*green*) and astrocytes stained red. The yellow signal is due to co-localization of the astrocytic marker GFAP (*red*) and IL-6 or CXCL8 (*green*) within astrocytes. **e** CCL2 (*green*) inside astrocytes stained red. **f, g** Evidence of IL-6 and CXCL8 (*green*) in microglia stained with anti-IBA1 antibody (*red*)

We focused on the cellular sources of IL-6, CXCL8, and CCL2. Interaction of *B. burgdorferi* with rhesus monkey brain parenchyma sections elicited IL-6 production by several types of glial cells, namely, astrocytes, oligodendrocytes, neurons, and microglia. CXCL8 and CCL2 were not found to be produced by neurons and microglia, respectively, but were present in the other cell types (Table 1 and Fig. 3).

**Table 1:** Phenotypes of immune mediator producer cells as visualized by confocal microscopy

	IL-6	CXCL8	CCL2
Neuron (NeuN)	+	−	+
Astrocyte (GFAP)	+	+	+
Oligodendrocyte (CNPase)	+	+	+
Microglia (IBA-1)	+	+	−

### Analysis of SP/NK_1_R interactions in primary cultures of rhesus dorsal root ganglia cells

#### Quantification of pro-inflammatory mediators

**Fig. 4:**
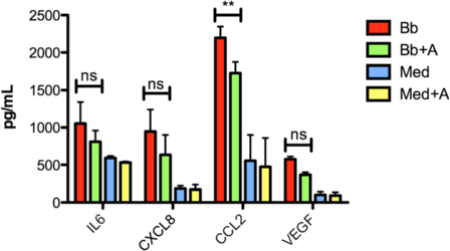
Pro-inflammatory mediator levels in supernatants from rhesus DRG cultures. IL-6, CXCL8, CCL2, and VEGF levels were detected by Multiplex ELISA in cells exposed to *B. burgdorferi* (Bb) or medium alone (Med), and treated with NK_1_R antagonist in the presence (Bb + A) or absence of *B. burgdorferi* (Med + A). The figure shows the arithmetic means ± standard deviations from two biological replicates. **p < 0.001, *ns* not significant

In previous studies, cells that were isolated from rhesus DRG and exposed to live *B. burgdorferi* in vitro, were shown to produce pro-inflammatory mediators, and DRG neurons were shown to undergo apoptosis [6]. To ascertain the effect of SP/NK_1_R interactions in cells of the PNS, we set up primary cultures of DRG cells from normal adult rhesus macaques and incubated the cultures with *B. burgdorferi* (MOI 10:1) for 24 h in the presence or absence of 10 μM NK_1_R antagonist. Results obtained with the Multiplex cytokine assay from two different biological replicates consistently showed that the interaction of *B. burgdorferi* with rhesus DRG cells elicited IL-6, CXCL8, CCL2, and VEGF. The addition of NK_1_R antagonist was able to significantly reduce the level of CCL2. The levels of the other mediators were also reduced, albeit not significantly (Fig. 4).

#### Quantification of apoptosis

**Fig. 5:**
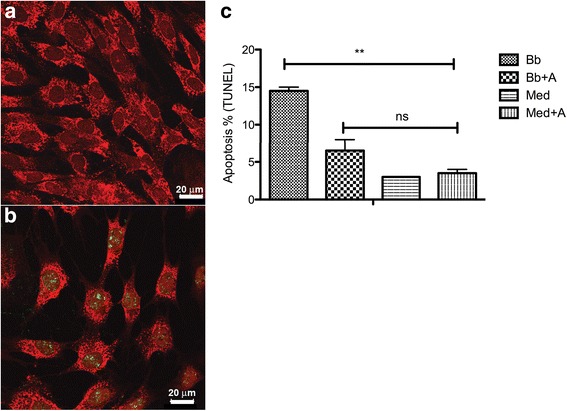
Apoptosis in DRG cell cultures. **a** Non-apoptotic field of neurons (NeuN-positive cells, *red*) cultured from rhesus DRG as visualized by fluorescent microscopy. **b** DNA fragmentation detected by the TUNEL assay (*green*) as visualized by fluorescent microscopy. **c** Graphical representation of the percent neuronal apoptosis as quantified by the TUNEL assay in cultured DRG cells exposed for 24 h to *B. burgdorferi* (Bb), exposed to *B. burgdorferi*, and treated with NK_1_R antagonist (Bb + A), in medium alone (Med), and medium plus antagonist (Med + A) (**p < 0.001)

*B. burgdorferi*-induced apoptosis of sensory neurons in the DRG cultures, and addition of the NK_1_R antagonist at a concentration of 100 nM significantly reduced the level of neuronal apoptosis (*p* < 0.001; Fig. 5).

## Discussion

Recently, using a NHP model of acute LNB, it was demonstrated that inflammation plays a key role in LNB pathogenesis [15]. Moreover, in a murine model, it was shown that SP, which is present throughout the CNS and is the most abundant tachykinin in the brain [16], increases inflammatory mediator production by astrocytes and microglia following exposure to either *Neisseria meningitidis* or *B. burgdorferi* [11]. These findings set the scene to investigate the immunoregulatory effects of SP/NK_1_R interactions in NHP cortical brain tissue and PNS neurons exposed to *B. burgdorferi*.

We have shown that co-incubation of both CNS tissues and PNS cells with the NK_1_R antagonist L703,606 attenuates bacterially induced increases in inflammatory cytokine and chemokine production, particularly IL-6, CXCL8, and CCL2 (Figs. 1 and 4), and reduces apoptosis levels of neural cells (Figs. 2 and 5). In addition, using confocal microscopy, we identified cellular sources of these immune mediators (Table 1 and Fig. 3) and confirmed that they are produced by glial cells and neurons [4]. These results suggest that NK_1_R antagonist treatment is able to reduce downstream pro-inflammatory signaling, thereby indicating that its systemic administration may slow down disease progression.

SP is a potent initiator of neurogenic inflammation in the CNS, an effect that is often followed by alterations in blood-brain barrier permeability and by persistent neurological deficits [10, 17]. The neuroinvasion that manifests in CNS neuroborreliosis depends on the successful translocation of spirochetes across the blood-brain barrier [18] and is concomitant with SP release [10]. We propose that the exacerbation of SP levels may accelerate disease progression, since blockage of NK_1_R in our ex vivo and in vitro models limits inflammation and improves cell survival. These results agree with the reported effects of NK_1_R antagonist treatment, which protected dopaminergic neurons, preserved barrier integrity, reduced neuroinflammation, and significantly improved motor function in a rat model of early Parkinson’s disease [19].

Results obtained from the interaction of *B. burgdorferi* with rhesus DRG cells indicate that activation of NK_1_R by SP can stimulate the chemokine CCL2. This chemokine plays a fundamental role in inflammation by recruiting inflammatory cells to specific sites [20], and we speculate that stimulation of CCL2 by SP can potentially mediate this recruitment in vivo. In addition, NK_1_R antagonist modestly reduced IL6, CXCL8, and VEGF production by DRG cells, following the same pattern as with CCL2. However, this phenomenon was not statistically significant (Fig. 4). SP induces a non-apoptotic form of cell death in hippocampal, striatal, and cortical neurons and thus plays an important role in pathological states in which neural cell death occurs [21]. Our results indicate that SP/NK_1_R interactions can also induce apoptotic cell death, as NK_1_R antagonist treatment prevented DRG neuronal apoptosis (Fig. 5). Taken together, these data suggest that the NK_1_R, in addition to modulating inflammation, may be a mediator of cell death in vivo.

We hypothesized that SP contributes to the pathophysiology of neuroborreliosis, and we evaluated this hypothesis with NK_1_R-expressing CNS tissues. Indeed, our results showed that both secreted and intracellular pro-inflammatory proteins were suppressed in the presence of NK_1_R antagonist (Fig. 1), albeit with animal-to-animal variation, in our ex vivo culture model of *B. burgdorferi* CNS infection. We also found an anti-apoptotic effect in oligodendrocytes that was mediated by the NK_1_R antagonist (Fig. 2), but not in neurons. Oligodendrocytes are vital for the functioning and survival of neurons, and the inflammation and subsequent apoptosis of oligodendrocytes induced by *B. burgdorferi* could contribute to the pathogenesis of LNB [1].

Previous studies have shown that NK_1_R signaling plays a role in numerous biological processes, such as the transmission of pain in the spinal cord [22, 23]. Activation of NK_1_R by SP leads to phosphoinositide hydrolysis, calcium mobilization, and mitogen-activated protein kinase (MAPK) activation [24, 25], and regulates neuroinflammation, neuronal survival, and synaptic activity [21]. Our results agree with this general picture, as they suggest that NK_1_R/SP interaction contributes to the development of *B. burgdorferi-*induced inflammation in the CNS and PNS. This interaction thus represents a therapeutic target for neuroinflammation.

From the pertinent literature, it is evident that SP is an important mediator of inflammatory responses and pathological conditions associated with inflammation, including rheumatoid arthritis, multiple sclerosis, inflammatory bowel disease, lymphatic contractility, and pneumococcal meningitis [26–30]. In our present model, inflammation mediated by SP/NK_1_R likely results from the synergistic effect of this interaction on the neural cell response to *B. burgdorferi*. We suggest that endogenous SP augments *B. burgdorferi-*associated pathology within the brain by elevating levels of inflammatory mediators and by promoting cell death probably though activation of the MAPK signaling cascade [31]. We have shown previously that the MEK/ERK pathway is crucial for *B. burgdorferi*-induced inflammation and P53-mediated apoptosis in oligodendrocytes [32].

The molecular mechanisms leading to inflammatory mediator release from the resident cells of the CNS when exposed to *B. burgdorferi* are numerous and are currently being investigated in our laboratory. Recently, several studies have shown that multiple receptors and pathways positively and negatively regulate microglial inflammation, resulting in a complex immune network [32–34]. CCL2/CCR2 and MAPK signaling, chiefly MEK/ERK, play a major role in neuroinflammation [32, 35], and our results are consistent with the possibility that blockage of NK_1_R may act by limiting these signaling cascades. Thus, in addition to antibiotics, treatment with NK_1_R antagonists could be explored as an adjuvant intervention against LNB.

## Conclusions

Our results indicate that NK_1_R antagonist treatment can attenuate bacterially induced increases of inflammatory mediator production in CNS and PNS cells, particularly, IL-6, CXCL8, and CCL2, as well as apoptosis levels.

## Abbreviations

CCL2: chemokine (C-C) motif ligand 2
CCR2: C-C chemokine receptor 2
CNPase: 2′, 3′- cyclic nucleotide 3′-phosphodiesterase
CNS: central nervous system
CSF: cerebrospinal fluid
DRG: dorsal root ganglia
FBS: fetal bovine serum
GFAP: glial fibrillary acidic protein
IFN-γ: interferon gamma
LNB: Lyme neuroborreliosis
MOI: multiplicity of infection
NeuN: neuronal nuclear protein N
NGF: nerve growth factor
P/S: penicillin and streptomycin
PBS: phosphate-buffered saline
PNS: peripheral nervous system
TUNEL: terminal deoxynucleotidyl transferase-mediated UTP nick end labeling
VEGF: vascular endothelial growth factor

## References

[1] Ramesh G, Benge S, Pahar B, Philipp MT (2012). A possible role for inflammation in mediating apoptosis of oligodendrocytes as induced by the Lyme disease spirochete *Borrelia burgdorferi*. J Neuroinflammation.

[2] Halperin JJ (2015). Nervous system Lyme disease. Curr Infect Dis Rep.

[3] Ramesh G, MacLean AG, Philipp MT (2013). Cytokines and chemokines at the crossroads of neuroinflammation, neurodegeneration, and neuropathic pain. Mediators Inflamm.

[4] Kondrusik M, Swierzbińska R, Pancewicz S, Zajkowska J, Grygorczuk S, Hermanowska-Szpakowicz T. [Evaluation of proinflammatory cytokine (TNF-alpha, IL-1beta, IL-6, IFN-gamma) concentrations in serum and cerebrospinal fluid of patients with neuroborreliosis]. Neurol Neurochir Pol. 2004;38:265–70. 15383953

[5] Ramesh G, Borda JT, Dufour J, Kaushal D, Ramamoorthy R, Lackner AA (2008). Interaction of the Lyme disease spirochete *Borrelia burgdorferi* with brain parenchyma elicits inflammatory mediators from glial cells as well as glial and neuronal apoptosis. Am J Pathol.

[6] Ramesh G, Santana-Gould L, Inglis FM, England JD, Philipp MT (2013). The Lyme disease spirochete *Borrelia burgdorferi* induces inflammation and apoptosis in cells from dorsal root ganglia. J Neuroinflammation.

[7] Myers TA, Kaushal D, Philipp MT (2009). Microglia are mediators of *Borrelia burgdorferi*-induced apoptosis in SH-SY5Y neuronal cells. PLoS Pathog.

[8] Vilisaar J, Kawabe K, Braitch M, Aram J, Furtun Y, Fahey AJ, Chopra M, Tanasescu R, Tighe PJ, Gran B, Pothoulakis C, Constantinescu CS. Reciprocal regulation of substance P and IL-12/IL-23 and the associated cytokines, IFNγ/IL-17: A perspective on the relevance of this interaction to multiple sclerosis. J Neuroimmune Pharmacol. 2015. 10.1007/s11481-015-9589-x PMC4543419 25690155

[9] Steinhoff MS, von Mentzer B, Geppetti P, Pothoulakis C, Bunnett NW (2014). Tachykinins and their receptors: contributions to physiological control and the mechanisms of disease. Physiol Rev.

[10] Rasley A, Marriott I, Halberstadt CR, Bost KL, Anguita J (2004). Substance P augments *Borrelia burgdorferi*-induced prostaglandin E2 production by murine microglia. J Immunol.

[11] Chauhan VS, Sterka DG, Gray DL, Bost KL, Marriott I (2008). Neurogenic exacerbation of microglial and astrocyte responses to *Neisseria meningitidis* and *Borrelia burgdorferi*. J Immunol.

[12] Roberts ED, Bohm RP, Lowrie RC, Habicht G, Katona L, Piesman J (1998). Pathogenesis of Lyme neuroborreliosis in the rhesus monkey: the early disseminated and chronic phases of disease in the peripheral nervous system. J Infect Dis.

[13] Philipp MT, Johnson BJ (1994). Animal models of Lyme disease: pathogenesis and immunoprophylaxis. Trends Microbiol.

[14] Pachner AR, Gelderblom H, Cadavid D (2001). The rhesus model of Lyme neuroborreliosis. Immunol Rev.

[15] Ramesh G, Didier PJ, England JD, Santana-Gould L, Doyle-Meyers LA, Martin DS (2015). Inflammation in the pathogenesis of Lyme neuroborreliosis. Am J Pathol.

[16] Marriott I (2004). The role of tachykinins in central nervous system inflammatory responses. Front Biosci.

[17] Turner RJ, Vink R (2014). NK1 tachykinin receptor treatment is superior to capsaicin pre-treatment in improving functional outcome following acute ischemic stroke. Neuropeptides.

[18] Mlynarcik P, Pulzova L, Bencurova E, Kovac A, Dominguez MA, Hresko S (2015). Deciphering the interface between a CD40 receptor and borrelial ligand OspA. Microbiol Res.

[19] Thornton E, Vink R (2012). Treatment with a substance P receptor antagonist is neuroprotective in the intrastriatal 6-hydroxydopamine model of early Parkinson’s Disease. PLoS One.

[20] Castellani ML, Vecchiet J, Salini V, Conti P, Theoharides TC, Caraffa A (2009). Stimulation of CCL2 (MCP-1) and CCL2 mRNA by substance P in LAD2 human mast cells. Transl Res.

[21] Castro-Obregón S, Del Rio G, Chen SF, Swanson RA, Frankowski H, Rao RV (2002). A ligand-receptor pair that triggers a non-apoptotic form of programmed cell death. Cell Death Differ.

[22] Quartara L, Maggi CA (1998). The tachykinin NK1 receptor. Part II: distribution and pathophysiological roles. Neuropeptides.

[23] Weisshaar CL, Winkelstein BA (2014). Ablating spinal NK1-bearing neurons eliminates the development of pain and reduces spinal neuronal hyperexcitability and inflammation from mechanical joint injury in the rat. J Pain.

[24] Rosso M, Muñoz M, Berger M (2012). The role of neurokinin-1 receptor in the microenvironment of inflammation and cancer. Sci World J.

[25] Bouzas-Rodríguez J, Zárraga-Granados G, Sánchez-Carbente Mdel R, Rodríguez-Valentín R, Gracida X, Anell-Rendón D (2012). The nuclear receptor NR4A1 induces a form of cell death dependent on autophagy in mammalian cells. PLoS One.

[26] Makino A, Sakai A, Ito H, Suzuki H (2012). Involvement of tachykinins and NK1 receptor in the joint inflammation with collagen type II-specific monoclonal antibody-induced arthritis in mice. J Nippon Med Sch.

[27] Reinke EK, Johnson MJ, Ling C, Karman J, Lee J, Weinstock JV (2006). Substance P receptor mediated maintenance of chronic inflammation in EAE. J Neuroimmunol.

[28] Nessler S, Stadelmann C, Bittner A, Schlegel K, Gronen F, Brueck W (2006). Suppression of autoimmune encephalomyelitis by a neurokinin-1 receptor antagonist—a putative role for substance P in CNS inflammation. J Neuroimmunol.

[29] Chakraborty S, Nepiyushchikh Z, Davis MJ, Zawieja DC, Muthuchamy M (2011). Substance P activates both contractile and inflammatory pathways in lymphatics through the neurokinin receptors NK1R and NK3R. Microcirculation.

[30] Chauhan VS, Kluttz JM, Bost KL, Marriott I (2011). Prophylactic and therapeutic targeting of the neurokinin-1 receptor limits neuroinflammation in a murine model of pneumococcal meningitis. J Immunol.

[31] Castro-Obregon S, Rao RV, del Rio G, Chen SF, Poksay KS, Rabizadeh S (2004). Alternative, nonapoptotic programmed cell death: mediation by arrestin 2, ERK2, and Nur77. J Biol Chem.

[32] Parthasarathy G, Philipp MT (2014). The MEK/ERK pathway is the primary conduit for *Borrelia burgdorferi-*induced inflammation and P53-mediated apoptosis in oligodendrocytes. Apoptosis.

[33] Ramesh G, Philipp MT, Vallières L, MacLean AG, Ahmad M (2013). Mediators of neuroinflammation. Mediators Inflamm.

[34] Parthasarathy G, Philipp MT (2015). Inflammatory mediator release from primary rhesus microglia in response to *Borrelia burgdorferi* results from the activation of several receptors and pathways. J Neuroinflammation.

[35] Maddahi A, Edvinsson L (2010). Cerebral ischemia induces microvascular pro-inflammatory cytokine expression via the MEK/ERK pathway. J Neuroinflammation.

